# Interactive social pragmatic intervention and responsive engagement (INSPIRE): An intervention program to facilitate social skills among toddlers with autism

**DOI:** 10.1016/j.mex.2025.103352

**Published:** 2025-05-03

**Authors:** Ramandeep Kaur

**Affiliations:** Professor and Head (Speech Pathology), Father Muller College, Department of Speech and Hearing, Mangalore, India

**Keywords:** Pragmatic language skills, Social pragmatic interventions, Responsive engagement, Autism spectrum disorder, Language Delays, Preschool Language Development, Social Skills Training, INSPIRE-Core

## Abstract

Pragmatic skills—how children use language in social situations—begin to develop early in life and are important for toddlers as they learn to communicate their needs, build relationships, and explore their environment. While many toddlers naturally pick up these skills through everyday interactions, there is growing recognition that some may benefit from early support. However, targeted intervention strategies for enhancing pragmatic development in toddlers are still not widely explored. The primary objective of this study was to develop a comprehensive intervention program designed to foster pragmatic skills among toddlers who are diagnosed with Autism. The overall process of development of this program, was conducted in three distinct phases. The first phase focused on creating various illustrative stories along with activities targeting specific pragmatic domains. In the second phase, an expert validation process was carried out, engaging a team of experienced speech-language pathologists, and other professionals along with parents of children with Autism. As a result, the study produced a Toolkit named *INSPIRE-Core* for toddlers between 1 and 3 years. The third phase focused on standardization of this toolkit by parental implementation of this program on 50 children with specific inclusion and exclusion criteria, within home environments. Overall, the study demonstrated that the intervention program served as an effective and structured resource for parents, supporting systematic planning and implementation of pragmatic language interventions.

• A three-phase design was employed to systematically develop and validate the intervention program, targeting pragmatic skills in children with language delays.
• Expert validation ensured the program's robustness, involving speech-language pathologists, other professionals and parents of children with autism.
• Standardization was achieved through implementation on a stratified sample of 50 children with autism, categorized by language age and trained within home environments.

## Graphical abstract

**Figure:**
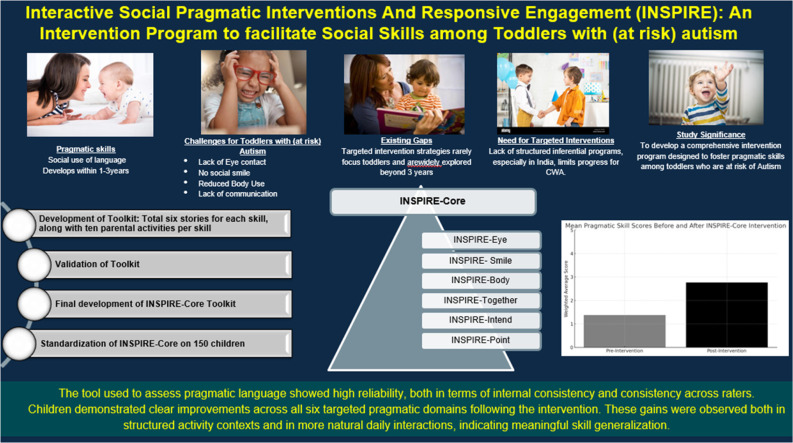

Specifications table

**Table:** 

Subject area:	Psychology
More specific subject area:	Speech Language Pathology
Name of your method:	INSPIRE-Core
Name and reference of original method:	Prizant, B. M. W., Amy M.; Rubin, Emily; Laurent, Amy C.; Rydell, Patrick J [5]. The SCERTS Model: A Comprehensive Educational Approach for Children with Autism Spectrum Disorders (Vol. 1). Baltimore, Md., USA: Paul H. Brookes
Resource availability:	The authors can make the data available upon a reasonable request. The treatment program will be published as a treatment manual, in future

## Background

Pragmatic skills refer to the social use of language which are foundational for toddlers to communicate needs, build relationships, and explore their environment. While these skills emerge naturally during typical development, children with autism spectrum disorder (ASD) often exhibit delays in core pragmatic behaviors like eye contact, joint attention, and pointing (Adams, [1]; Baron-Cohen et al., [3]). Such delays can cascade into socialization and language acquisition challenges, underscoring the need for early intervention.

However, structured programs targeting *foundational* pragmatics in toddlers are scarce, particularly in culturally adaptable formats. The INSPIRE-Core program addresses this gap by providing a systematic toolkit to nurture six key skills: eye contact, smiling, body posture, joint attention, communication intent, and pointing through storybooks and 60 scaffolded activities.

### Motivation for methodology

A key driver for developing the INSPIRE-Core methodology was the developmental urgency associated with early pragmatic deficits in toddlers with autism spectrum disorder (ASD). Research has shown that delays in foundational social-communication behaviors—such as eye contact, pointing, and joint attention—can persist over time and lead to broader difficulties in language acquisition and social interaction (Geurts et al., [4]). Recognizing the neuroplastic potential during the critical 1–3-year developmental window, INSPIRE-Core was intentionally designed to intervene early, when children are most responsive to structured yet naturalistic input.

Equally important was the need for a structured yet play-based approach that would break down complex pragmatic skills into teachable units. Unlike broader communication programs such as *More Than Words* (Sussman, [6]), INSPIRE-Core targets six specific skills through 60 clearly graded activities supported by narrative storybooks. This format strikes a balance between structured repetition and playful interaction, making it easier for children to understand, retain, and apply new skills across everyday routines. The scaffolded nature of the activities also allows caregivers to progress gradually while reinforcing mastery at each step.

In designing the toolkit, special attention was given to cultural adaptability. Existing models like SCERTS® (Prizant et al., [5]), while comprehensive, often reflect Western social norms and assumptions about interaction styles—such as expectations of prolonged eye contact—that may not align with all cultural contexts. INSPIRE-Core was reviewed and validated by a team of Indian speech-language pathologists, other professionals and caregivers, ensuring that the strategies are sensitive to local customs, beliefs, and caregiver–child interaction patterns. This makes the toolkit more accessible, acceptable, and effective within the Indian socio-cultural framework.

Finally, the method was deliberately structured to promote caregiver empowerment. The intervention does not rely on intensive clinical supervision or specialized tools; instead, it uses simple activity prompts, visual support materials, and progress-monitoring checklists that enable parents to confidently deliver sessions within the home environment. This caregiver-led model helps bridge gaps in access to therapy, especially in under-resourced settings or very early stages of child development. The program also reinforces the parent–child relationship as a primary context for language and social development.

### Methodological foundations

The program integrates:

• Speech-Act Theory (Austin, [2]): Teaches toddlers to link gestures (e.g., pointing) to communicative intent (*illocutionary acts*).
• Theory of Mind (Baron-Cohen et al., [3]): Activities like joint attention (*INSPIRE-Together*) foster perspective-taking.
• Naturalistic Developmental Behavioral Interventions (NDBIs): Skills are embedded in play routines to mimic real-world interactions.

### Validation and standardization

The INSPIRE-Core program underwent rigorous development in three phases to ensure efficacy and cultural appropriateness.

a. *Toolkit Creation* phase involved designing age-appropriate stories and 60 skill-specific activities (e.g., eye contact, joint attention) with input from speech-language pathologists.
b. *Expert Validation* engaged a team of experienced Indian speech-language pathologists, and other professionals along with parents of children with Autism to evaluate content clarity, cultural relevance, and practicality using a 5-point scale; only items rated ``Good'' or higher were retained, while others were refined or removed.
c. *Standardization Phase* involved implementing the toolkit with 50 toddlers with ASD (language age 6 months–2.11 years) across home settings, yielding excellent reliability (test-retest *r* = 0.91) and confirming its effectiveness for real-world use.

### Method details

The present study aimed to evaluate the effectiveness of the *INSPIRE-Core* caregiver-mediated intervention program in enhancing foundational pragmatic language skills in toddlers with autism spectrum disorder (ASD). The specific objectives were:

1. To develop *INSPIRE-Core* intervention program consisting of various activities and stories to guide parents to address six foundational pragmatic skills (eye contact, social smiling, body posture, joint attention, communication intent, and pointing) at home setting, under the guidance of Speech language pathologists.
2. To validate the *INSPIRE-Core* intervention program through an expert review system
3. To conduct a pilot study on children with Autism to establish standardization and reliability

### Study design and setting

The study adopted a three-phase, mixed-methods design, combining qualitative and quantitative approaches. Phase 1 involved *INSPIRE-Core* intervention program development, Phase 2 involved expert validation, and Phase 3 was a quasi-experimental pre-test/post-test trial for standardization. The intervention program was designed for home-based caregiver delivery, with professional supervision from speech language pathologists and periodic follow-up. The families were recruited from Speranza, a Multi-speciality therapy center located in Karnataka, India. Regular implementation fidelity checks, and virtual assessments were conducted under the supervision of licensed speech-language pathologists.

### Phase 1: intervention development

The first phase focused on developing the toolkit, a culturally contextualized intervention manual designed to improve pragmatic language skills in toddlers between 1 and 3 years of age. This toolkit included:

• Six core domains targeting foundational social-communication skills: Eye Contact, Social Smiling, Body Posture, Joint Attention, Communication Intent, and Pointing.
• For each domain, 10 structured activities were developed (total = 60), intended to be embedded into everyday routines.
• Six illustrated short stories were written to introduce and model each skill in a relatable, child-friendly way.

The materials were drafted in consultation with a team of child development experts, which included Speech language pathologists, child psychologists, and developmental paediatricians, all of who were working in paediatric set-up for a minimum of 5 years. After the activities and stories were developed, illustrations were hand-drawn by a professional artist using age-appropriate visuals and culturally familiar scenarios. The illustrations for all stories and activities were not only child-specific but were also designed to be simple, engaging, and implementable by caregivers in home settings.

The first version of tool thus provided a story book and an activity book containing 10 ideas to elicit each targeted skill, totalling 60 activities. These activities were tailored to develop basic social and communicative skills. The series focused on the fundamental aspects of early social and communicative development. It lays the groundwork for children to understand and engage in basic social interactions:

1. **INSPIRE-Eye:** Emphasizes the importance of eye contact as a foundational element of communication and social interaction.
2. **INSPIRE-Smile:** Encourages smiling to express friendliness and positive emotions.
3. **INSPIRE-Body:** Focuses on appropriate body posture to convey interest and attentiveness.
4. **INSPIRE-Together:** Introduces joint attention as a key skill for shared experiences and communication.
5. **INSPIRE-Intent:** Develops an understanding of communication intention, helping children grasp the purpose behind interactions.
6. **INSPIRE-Point:** Teaches the use of pointing as a basic yet effective means of non-verbal communication.

### Phase 2: content validation

In the second phase, an expert validation process was undertaken to ensure the toolkit’s clarity, appropriateness, and usability.

• **Participants in validation:**
  A total of 12 experts participated in this phase, all who had a minimum of 5years of experience in paediatric population. The experts include:
  ○ Four Certified speech-language pathologists (SLPs)
  ○ Two child psychologists
  ○ Two paediatricians
  ○ One occupational therapist-paediatrics
  ○ One Montessori teacher and special educator
  ○ Two Parents of toddlers with ASD
• **Procedure:** Each expert was asked to review all stories, activities, and illustrations drawn, using a structured rating scale with five criteria:
  1. Simplicity of content of stories and activities
  2. Familiarity and cultural relevance of the of stories and activities
  3. Illustrations drawn (culture-specificity, familiarity of pictures, picture size)
  4. Stimulability and generalizability of each of the stories and activities
  5. Overall usability and trainability of the toolkit
• **Scoring and Revisions:**Items were rated on a 5-point scale: *Very Poor, Poor, Fair, Good*, and *Excellent*.
  ○ Items rated “Good” or “Excellent” were retained as-is.
  ○ Items rated “Poor” or “Fair” were revised based on qualitative feedback from experts.
  ○ Any items rated “Very Poor” were removed entirely.

After these adjustments, a final review was conducted, and Table 1 reflects this process, with items achieving Fair, Good and Excellent as scores. The meticulous review process highlights the quality and efficacy in addressing the specific needs of children concerning the development of social and pragmatic skills.

**Table 1:** Expert ratings of INSPIRE-core toolkit parameters during validation phase.

S. No.	Parameter	Very poor	Poor	Fair	Good	Excellent
1	Simplicity of the tool	–	–	2	4	6
2	Familiarity of the tool	–	–	3	6	3
3	Size of pictures	–	–	1	5	6
4	Color and appearance	–		–	8	4
5	Relevance of stories and pictures in real life	–		1	8	3
6	Trainability of tool	–	–	1	5	6
7	Accessibility of tool	–	–	1	9	2
8	Flexibility of tool	–	–	–	3	9
9	Stimulability of tool	–	–	1	3	8
10	Generalization	–	–	–	8	4

After this iterative validation process, the toolkit was finalized and named INSPIRE-Core, indicating its role in nurturing core pragmatic competencies in toddlers. The validated version included instructions for caregivers, illustrations, scoring templates, and structured guidance for home-based delivery.

### Phase 3: standardization of INSPIRE-Core

#### Participants

An initial informal online screening was conducted with 150 children under the age of 3 years, using parent interviews and symptom checklists. Out of these, 80 children were flagged as showing signs of social delay, red flags for autism, or emerging pragmatic communication issues.

These 80 children, along with 13 others referred clinically (total *n* = 93), underwent individualized in-person screening using the Modified Checklist for Autism in Toddlers, Revised with Follow-Up (M-CHAT R/F). The M-CHAT R/F is a widely used, validated screening tool for early identification of ASD symptoms (Robins, Fein & Barton, 2009). A score of greater than 3 on the M-CHAT R/F was considered a positive screen warranting further diagnostic evaluation.

• Of the 93 children screened, 70 scored above 3, and 13 scored ≤ 3.
• The 13 children with lower scores were not immediately enrolled but advised for 6-month follow-up monitoring due to mild or ambiguous signs.
• The 70 children who screened positive were further referred to a licensed Speech-Language Pathologist for diagnostic confirmation using the Childhood Autism Rating Scale (CARS-2).

The CARS-2 assessment was used to categorize ASD severity:

• 62 children were rated as having mild-to-moderate autism, based on total CARS-2 scores between 30 and 36.5.
• 5 children scored above the severe autism threshold and were referred for intensive speech and occupational therapy.
• 3 children with borderline ASD features (CARS-2 scores between 28 and 30) were referred for weekly therapy sessions and were not included in the current intervention.

**Fig. 1:**
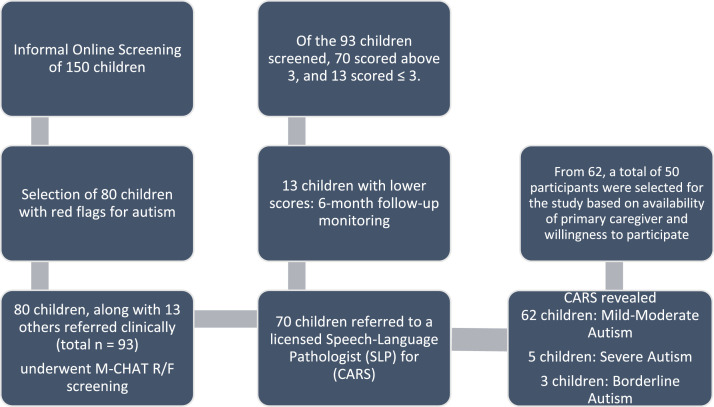
Overview of the recruitment process for INSPIRE-core study design.

Based on the sample size requirements, out of these 62 children, a total of 50 participants were selected based on factors like availability of primary caregiver, willingness to participate and so on. A summary of this process of recruitment is provided in Fig. 1.

### Sample size estimation

The required sample size was calculated using the formula for estimating a sample mean difference in paired observations:

$$n={\left(\frac{Z_{\alpha /2}+Z_{\beta }}{\Delta /\sigma }\right)}^{2}$$

Where:

• $Z_{\alpha /2}=$ 1.96 for a 95% confidence lavel
• $Z_{\beta }=$ 0.84 for 80% Power
• Δ= expected mean difference in post-intervention scores (estimated from pilot date = 1.2)
• σ= standard deviation of the difference (from pilot = 2.0)

This yielded a minimum required sample of 44 participants. To account for a 10–15 % dropout rate, a final sample of 50 toddlers was targeted and successfully enrolled.

The final sample for this study thus consisted of 50 toddlers with mild-to-moderate ASD.

### Inclusion criteria

• Chronological age between 12 and 36 months
• Confirmed diagnosis of mild-to-moderate autism spectrum disorder based on clinical assessment and CARS-2 scores.
• Availability of a consistent caregiver (parent or grandparent) who could participate in training and deliver the home-based intervention.
• Minimum receptive language and attention span necessary to participate in play-based, structured tasks.

### Exclusion criteria

• Children with significant intellectual disability, as indicated by developmental assessments showing global delays across domains.
• Uncontrolled behavioral issues (e.g., aggression, self-injury, extreme non-compliance) that could compromise the home setting intervention.
• Major uncorrected sensory or motor impairments, including visual, auditory, or neuromotor disabilities interfering with communication or task execution.

### Demographics and consent

Among the 50 participants, ages ranging from 12 to 36 months (*M* = 24.3 months, SD = 4.8). There were 30 girls and 20 boys, and most children were early communicators (using gestures, vocalizations, or single words). The primary implementers of the intervention were mothers (80 %), fathers (15 %), and grandparents (5 %).

All caregivers signed informed consent forms after receiving a thorough explanation of the study procedures, ethical guidelines, and confidentiality provisions. The study was approved by the Father Mullers Institutional Review Board.

### Intervention procedure

The INSPIRE-Core intervention was systematically implemented across four interrelated phases: [] Pre-Intervention Assessment [2], Intervention Delivery [3], Post-Intervention Evaluation, and [4] Maintenance and Follow-Up. Each phase was designed to ensure comprehensive assessment, skill-focused caregiver training, skill acquisition through structured home routines, and sustainability of gains over time. A combination of standardized tools, observational measures, and caregiver reports was employed to document outcomes.

### Phase I: pre-intervention assessment

The initial phase involved the baseline assessment of each child’s pragmatic communication skills, conducted to establish a clear pre-intervention profile and guide individualized implementation. A validated, domain-specific observational checklist was administered on children and used as a baseline outcome measure. The checklist included 12 items distributed across the six foundational domains targeted in the INSPIRE-Core program:

• Eye Contact
• Social Smiling
• Body Posture
• Joint Attention
• Communication Intent
• Pointing

Each item was scored on a five-point ordinal scale, ranging from:

1 = Never

2 = With Assistance

3 = Sometimes with Verbal Prompts

4 = Mostly Independently

5 = Always Independently

This scale captured the degree of skill independence and contextual generalization. Items such as “My child initiates and maintains eye contact during interactions” and “My child adjusts their communication based on the listener’s response or feedback” allowed for both caregiver insight and clinician validation through structured observation.

In addition to checklist completion, a brief semi-structured interview was conducted with caregivers to gather contextual data on the child’s daily communication behavior, exposure to prior intervention, and caregiver expectations. This phase ensured the systematic documentation of each participant’s baseline functioning and readiness for structured input.

### Phase II: intervention delivery

The second phase constituted the core implementation period, spanning 6 months. During this period, caregivers (primarily parents) were the primary agents of intervention delivery, following structured guidelines and materials provided in the INSPIRE-Core toolkit. Intervention was implemented three times per week in the child’s naturalistic home environment, for 20–30 min per session.

### Parental training and capacity building

Prior to implementation, caregivers participated in a 2-hour structured training module facilitated by certified speech-language pathologists. The training was divided into two key components:

• Activity Demonstration and Discussion:
  ○ 45 min: Introduction and modeling of domain-specific play-based activities.
  ○ 15 min: Group discussion on strategies for engagement, prompting, and individualized adaptation.
• Shared Book Reading Module:
  ○ 45 min: Demonstration of evidence-based shared book reading techniques using six storybooks aligned with each targeted domain.
  ○ 15 min: Collaborative discussion on generalization and story customization.

Training sessions emphasized interactive modeling, guided role-play, errorless teaching techniques, and caregiver reflection. Caregivers received a printed activity manual, visual aids, sample props, and a session logbook to document home-based sessions. Fidelity to training content was reinforced through fortnighly check-in meetings and weekly telephonic coaching..

### Structure of home-based sessions

Each intervention session followed a semi-structured yet adaptable format:

• Warm-Up (5 min): Review of a previously targeted skill in a motivating context.
• Skill-Focused Activity (15–20 min): Implementation of 1–2 structured play routines targeting the weekly skill (e.g., “Look here!” games for eye contact, pointing to snack items for requesting).
• Generalization Segment (5 min): Application of the same skill during daily routines (e.g., brushing, mealtime, or play with siblings).
• Reinforcement: Positive feedback strategies such as verbal praise, clapping, preferred toys, or tangible rewards.

Caregivers completed a session log after each interaction, noting child responsiveness, engagement level, and any contextual adaptations. Fortnightly in-person follow-ups were conducted to monitor progress and weekly coaching was provided on telephone.

The intervention was structured across a 6-month timeline, with each of the six foundational pragmatic skills addressed in one dedicated month. This approach allowed for deep engagement, focused repetition, and gradual skill acquisition within developmentally appropriate timeframes.

Each month focused exclusively on one of the six target domains—Eye Contact, Social Smiling, Body Posture, Joint Attention, Communication Intent, and Pointing—ensuring comprehensive coverage of all skills by the end of the intervention phase. To understand this better, Table 2 provides an overview of INSPIRE-Core Program.

**Table 2:** Sample of INSPIRE-core activities and stories.

Skill	Description	Activities	Story
**INSPIRE-EYE**	Eye contact is a vital skill in communication and social interaction, serving as a key indicator of attention, interest, and engagement. For children, developing strong eye contact is foundational for building relationships, expressing emotions, and understanding social cues. As a parent, you play a crucial role in nurturing this skill. This section will provide you with insights and strategies to support your child in enhancing their eye contact, fostering better communication and social connections.	**1. Face Painting:** Set up a face painting station with washable paints and mirrors. • As you paint your child's face, maintain eye contact and encourage them to look at you by asking them to choose colors or designs. • Offer to let your child paint your face, guiding them to make eye contact as they work. **2. Silliness with Props:** • Gather silly props like clown noses, funny glasses, or stick-on mustaches. • Take turns wearing the props and making funny faces at each other. • Encourage your child to look into your eyes as you both laugh and enjoy the silliness. **3. Stickers on Face:** • Place stickers or googly eyes on your face, starting further away from your eyes. • Gradually move the stickers closer to your eyes, encouraging your child to follow them with their gaze. • Engage in a conversation or make funny expressions to keep your child's attention on your eyes	Page 1: "Hello! I'm Lila. Let's have fun with our eyes today." Page 2: "When Mommy says 'Peekaboo,' I look up. We laugh and our eyes sparkle together." Page 3: "Daddy winks and I try to wink back. It's like we're talking, just with our eyes." Page 4: "Here comes my puppy, his eyes are bright. We look at each other and feel so right." **………..and so on.**
**INSPIRE-SMILE**	Social smiling is a fundamental aspect of human interaction that conveys warmth, friendliness, and approachability. It is one of the earliest forms of communication and plays a crucial role in forming connections and expressing emotions. For children, developing a social smile is an essential step in learning how to interact with others, even before language skills are fully developed.	1. **Mirror Play:** • Sit with your child in front of a mirror. • Make funny faces and smile widely, encouraging your child to imitate you. • Celebrate when they mimic your smile by clapping or cheering, reinforcing the behavior. 2. **Smile and Sing:** • Incorporate smiles into nursery rhymes and songs. • Sing songs that have actions, including smiling, and use a puppet or a toy that smiles. • Make it a game to smile every time a certain word is sung. **3. Emotion Matching Game:** • Create cards with pictures of people displaying different emotions, including happy and agreeable expressions. • Have your child match expressions with appropriate scenarios (like receiving a gift or playing with a friend). • Prompt your child to mimic the happy and agreeable expressions, reinforcing the connection between the emotion and the smile. **4. Interactive Games with Peers:** • Arrange social play time with peers and play interactive games that involve turn-taking and greeting, like "Duck, Duck, Goose" or "Musical Chairs". • Encourage the children to smile and say "hello" or "good job" to each other during the game.	Page 6: Smiles are warm, like the sun's glow, making everyone happy, high and low! Page 7: Let's smile at everyone we see. Smiles for you, smiles for me, spreading joy, so free! Page 8: Smiles are like sunshine, making hearts feel bright. They turn every moment into a joyful, loving sight. **………..and so on.**
**INSPIRE-BODY**	Body posture is not just about physical stature; it's a powerful way to perceive and interact with others. This is especially crucial for language-delayed children, as posture can bridge communication gaps and convey emotions and intentions when words are limited.	**1. Puppet Show Interactions:** • Set up a puppet show where the puppets demonstrate good and poor listening postures. • Ask your child to help the puppet that is not showing good posture to sit up straight and look at the speaker. • Give the child a turn to manipulate a puppet and practice good listening posture. **2. Musical Statues with Postures:** • Play musical statues, but instead of freezing in any pose, they must freeze in an attentive posture. • Define the attentive posture as standing or sitting up straight, with eyes looking forward, and hands still. • Music and movement make it fun, and the pause helps practice the posture. **3. Teddy Bear Picnic:** • Have a picnic with your child and their teddy bears or other stuffed animals. • Assign each bear a "listening spot" where they sit up straight and face whoever is 'talking'. • Encourage your child to adjust the bears to sit attentively and then do the same themselves.	Page 3: Farah the Flamingo, pink and light, stands on one leg, oh what a sight! Mina tries too, and starts to giggle, standing on one foot, just a little. Page 4: Ellie the Elephant, big and strong, lifts her trunk all day long. "Keep your back straight, hold your head high," she says with a happy, loud sigh. Mina tries it and feels like she can fly! **………..and so on.**
**INSPIRE-JOINT**	Joint attention is a critical social skill where a child and another person focus together on an object or event, paving the way for language and social development. Parents can foster joint attention by following their child's gaze or pointing gestures, then verbally labeling the object of focus.	1. **Bubble Play:** • Blow bubbles and encourage your child to watch where they go, pointing and looking together. • Take turns blowing and chasing the bubbles, using language like "Look!" and "See the bubble?" to direct attention. 2. **Light-up Toy Play:** • Use a toy that lights up or makes sounds when pressed. • Press the button to activate the toy, then wait for your child to look at the toy before pressing it again. • This activity helps them learn to follow your gaze and share attention on the toy. 3. **Painting Together:** • Share a large sheet of paper and paint together. • Point out each other's colors and strokes, saying things like "Look, I'm using blue!" and wait for your child to show you their painting. • This activity encourages joint attention through shared creative expression.	Page 1: Twinkle, twinkle, little stars, with Papa, Aashu reaches far! Page 2: "See the moon, Aashu?" Papa points up high. Aashu looks and claps, under the night sky. Page 3: They peek through a small book folded, round and neat. "Stars are twinkling," Aashu giggles, "Look, how they tweet!" Aashu, eyes sparkling with light. **………..and so on.**
**INSPIRE-INTENT**	Communication intent refers to the purpose or reason behind a person's attempt to communicate. It is the underlying motivation driving an individual to express thoughts, needs, feelings, or information. In early childhood, understanding and recognizing communication intent is crucial for language development and social interaction.	1. **Mealtime Choice Board:** • Create a choice board with pictures of different foods. • During mealtime, present the board to the child. • Let the child point to the picture of the food they want to eat. 2. **Activity Choice Board:** • Make a choice board with pictures of various activities (e.g., drawing, playing with blocks, reading a book). • Show the board to the child and explain the options. • Allow the child to choose their preferred activity by pointing to the picture. • Similar choice boards can be used during the activity to denote next steps or required items in an activity **Modeling and Prompting:** 1. **Requesting a Snack:** • Hold a snack and say "I want a snack." • Encourage the child to repeat the phrase. • If the child gestures or vocalizes, provide the snack as reinforcement.	Page 5: Ribbit the Frog croaks loud and clear. Tara tries too, without any fear. "Rib-rib, bit-bit," she sings in the rain, talking makes her so happy again. Page 6: Night falls, and Tara whispers, "Night-night." Moon smiles down with a soft, gentle light. "Tomorrow more words," Mama says with a kiss, "In your tiny talks, there's so much bliss." **………..and so on.**
**INSPIRE-POINT**	Pointing is more than just a simple gesture; it's a cornerstone of early communication. It's one of the first ways children express their interests, needs, and desires to others.	1. **Modeling:** Start by modeling the behavior you want to see. For example, when you want to show your child something, point to it and say its name clearly. Make sure to do this consistently to help your child understand the connection between pointing and verbalizing. 2. **Guided Practice:** Guide your child's hand to point to objects while simultaneously encouraging them to say the name of the object. You can say, "Let's point to the ball and say 'ball.'" This helps to associate the act of pointing with verbalizing. 3. **Use Visual Aids:** Visual aids such as picture cards or flashcards can be helpful. Show your child a card, point to the picture, and say the word together. Gradually encourage your child to take the lead in pointing and saying the word.	Page 5: Manu points up high, "Birds!" he says with a shout. He loves pointing things out. Page 6: Laila the Parrot flies down. She points with her wing. "Look, bananas!" says Laila, doing a swing. **………..and so on.**

The first session of each month (Week 1, Session 1) began with the introduction of a domain-specific storybook, designed to model the target skill in a relatable and child-friendly narrative. During this session, caregivers were encouraged to read the story aloud, use visual referencing, and prompt the child’s attention to key social-communication cues illustrated in the narrative (e.g., shared attention, smiling back, pointing, etc.).

For the remaining two sessions of Week 1 and all three sessions in subsequent weeks, caregivers selected from the 10 structured play-based activities aligned with the month’s target skill. These activities emphasized hands-on engagement and functional use of the skill within naturalistic home environments. The parents were free to choose from any activity provided they worked on each activity atleast once during the month.

Each new week within the month began with a brief review of the same storybook, reinforcing conceptual familiarity and ensuring continuity. Following this review, caregivers proceeded with one or more activity-based tasks designed to scaffold the child’s use of the targeted pragmatic behavior.

This weekly cycle—*story review followed by activity implementation*—was repeated across the four weeks of each month. The repeated exposure to a single skill across three structured sessions per week, supported by visual and interactive narratives, was intended to promote consolidation, generalization, and retention of pragmatic behaviors.

Caregivers recorded session-specific details in a logbook, including:

• The activity or story reviewed
• Child’s responsiveness and level of independence
• Engagement time and attentional span
• Any contextual adaptations or challenges faced

These logs were reviewed bi-weekly by the research team to monitor fidelity and track progress. Structured follow-up calls and in-home video observations, as detailed previously, further supported consistency across families and ensured alignment with the intervention framework.

### Troubleshooting and individual adaptation

To accommodate variability in child response and temperament, caregivers were trained to implement gentle prompting hierarchies, starting with high support (e.g., hand-over-hand) and gradually fading to independent performance. For children with slower progress, troubleshooting strategies from the manual were employed—for example:

• For limited eye contact: initiating short face-to-face games like peek-a-boo
• For difficulty with pointing: modeling the gesture and physically guiding the hand

**Fig. 2:**
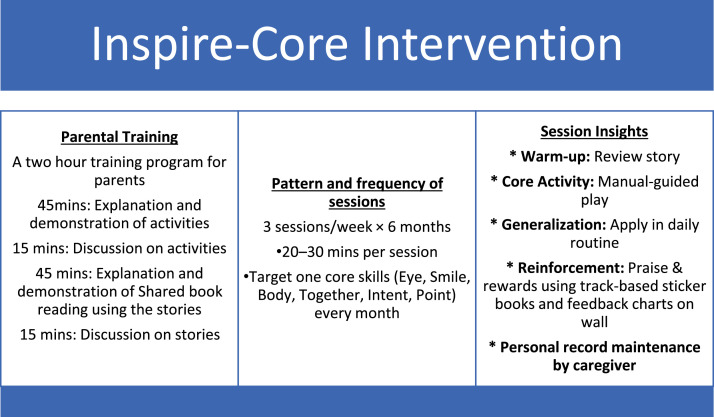
Structure of the INSPIRE-core intervention.

Conversely, children who showed rapid mastery of a skill were given more complex variations or tasked with using the skill across unfamiliar contexts to ensure generalization. An overview of the intervention program is provided in Fig. 2.

### Phase III: post-intervention evaluation

Following completion of the intervention period, participants underwent a comprehensive post-intervention assessment to evaluate progress in the six targeted pragmatic skill areas and gather caregiver feedback.

• **Checklist *Re*-Administration:** The same 12-item INSPIRE pragmatic skills checklist used during pre-intervention was re-administered. Parents scored each item, reflecting the child’s current level of performance across social-communication contexts. Pre- and post-scores were later compared to quantify measurable change. A brief overview of the checklist is provided in Table 3.

**Table 3:** A 12-item INSPIRE-Core pragmatic skills checklist.

S.no	Questions	1 Never	2 With assistance	3 Sometimes Independently (verbal prompts)	4 Mostly independently	5 Always independently
1	My child initiates and maintains eye contact during interactions.					
2	My child maintains appropriate eye contact during conversations.					
3	My child uses smiling as a greeting or in response to social interactions.					
4	My child smiles appropriately to express happiness or agreement.					
5	My child faces the speaker with an open and attentive body posture.					
6	My child maintains appropriate physical posturing while engaging in conversation.					
7	My child shows objects to ensure the conversational partner is looking at the same object.					
8	My child maintains joint attention when engaged in activities or play.					
9	My child communicates with a clear intention, such as requesting or informing.					
10	My child adjusts their communication based on the listener’s response or feedback.					
11	My child uses pointing to indicate objects or locations during communication.					
12	My child understands and responds to pointing gestures made by others.
• **Clinical Follow-Up Session:** Each child was invited for a 45-minute follow-up session at the clinic, facilitated by a blinded speech language pathologist. The session included:
  ○ Informal, play-based tasks designed to elicit each of the six pragmatic skills.
  ○ Spontaneous interaction opportunities with the clinician and caregiver to assess generalization and natural use of skills.
  ○ Observation and rating of skill presence, consistency, and contextual appropriateness.
• **Caregiver Feedback and Reflection:** Following the completion of the intervention phase, caregiver perspectives on the toolkit were gathered using a structured feedback form and a brief interview session. The feedback form included both quantitative Likert-scale items and open-ended questions, designed to evaluate the usability, clarity, and perceived effectiveness of the INSPIRE-Core materials.

Additionally, a short semi-structured interview was conducted with each caregiver to elicit more in-depth reflections on:

• Changes observed in the child's pragmatic behaviors
• Ease of implementing activities within daily routines
• Usefulness of the storybooks and visual materials
• Suggestions for improving the toolkit’s content or delivery model

While the formal intervention sessions were ending, parents were encouraged to continue using the learned techniques during routine interactions (for example, keep reading the INSPIRE stories as bedtime stories, or integrate the social games into playdates). This conversation helped transition families into the next phase, stressing that continued practice could help maintain the gains.

### Phase IV: maintenance and follow-up

During this phase, no new intervention content was introduced and there were no scheduled weekly sessions. Instead, families were instructed to *maintain* and generalize the skills the child had learned. Caregivers were asked to naturally incorporate the targeted pragmatic skills into daily routines and play, using the strategies from INSPIRE-Core when opportunities arose. For example, they would prompt eye contact during meals (“Look at me when I say ‘yummy’!”), encourage joint attention when playing outside (“Can you show me the aeroplane in the sky?”), or wait for the child to use a gesture/word before responding to requests, as they had practiced. Essentially, the parents served as facilitators of ongoing practice in an unstructured format, ensuring the child didn’t lose the new abilities.

To support families during maintenance, the team provided a simple calendar with monthly tips (e.g., a suggested family activity each month that would involve one or more target skills, like a visit to the zoo to practice joint attention by pointing out animals). No formal therapy sessions were held, but a researcher made a weekly phone check-in to each family to see if the child was continuing to use the skills and if the parents had any concerns. These calls were brief (10–15 min) and served to reinforce the caregivers’ efforts (“It’s great to hear she’s waving hi to neighbours now—keep encouraging that!”) and to remind them of the upcoming follow-up assessment. If a family noted any regression or difficulties, an optional booster session was offered: in a few cases, the parents and child visited the center for a single coaching session to troubleshoot issues that arose during the maintenance period (for instance, one child had stopped pointing, so they revisited a favorite pointing game to re-engage him). This happened with five out of fifty participants.

At approximately 2 months from the post-intervention, a maintenance follow-up assessment was conducted for each child. This assessment was identical in format to the post-test: the evaluator (the same person who did the post-test, when possible) interacted with the child in play scenarios and completed the 0–2 ratings for each skill domain. The purpose was to determine whether gains observed at post-test were sustained over time. Caregivers also updated the team on any other interventions or preschool programs the child may have started in the interim, so that any external influences could be noted. Two out of fifty participants joined group therapy sessions weekly in a Speech therapy center and five out of these participants joined evening children's club to further fecilitate social skills. The follow-up scores were recorded on the scoring sheet, and qualitative notes were taken, especially if a skill had regressed (e.g., “eye contact less consistent than at post-test, possibly due to new daycare environment” as reported by parent). After this final evaluation, families received a summary report of their child’s progress across all three time points (Pre, Post, Maintenance), and were given referrals or recommendations for any next steps (such as more advanced social skills groups if appropriate). The study team thanked the families for their participation and concluded their formal involvement at this stage.

To evaluate the retention of skill gains, this phase considered:

• **Parental Monitoring:** Caregivers were encouraged to continue using strategies embedded in daily routines. At follow-up, they were asked to report whether each of the six domains was still evident in spontaneous interaction.
• Speech Pathologist **Check-In:** A brief follow-up session (similar to post-intervention) was conducted by the same Speech Pathologist. Skills were re-assessed informally using familiar tasks to detect any signs of regression or maintenance.
• **Support Recommendations:** Based on the follow-up findings, families were provided with personalized recommendations, which included suggestions for advancing to more complex social communication modules or maintaining current routines.

### Data analysis

All collected data were analyzed using IBM SPSS (Version 25) with an aim to evaluate the efficacy of the INSPIRE-Core intervention and document caregiver feedback. Quantitative analyses focused on the pragmatic skill ratings from the standardized scoring sheets. For each of the six skill domains, as well as the total composite score, descriptive statistics (mean, standard deviation, and range) were computed at Pre-Intervention (baseline), Post-Intervention, and Maintenance follow-up. These summary statistics were used to characterize the sample’s initial skill levels and the extent of change after intervention. Data inspection confirmed that scores were approximately normally distributed and free of significant outliers, justifying the use of parametric tests.

The primary efficacy evaluation compared baseline and post-intervention scores. A series of paired sample *t*-tests (two-tailed) was conducted, one for each skill domain and one for the total score, to determine whether mean post-test ratings were significantly higher than pre-test ratings. For instance, a *t*-test examined whether the average Eye Contact score improved from pre- to post-intervention. Given the relatively small number of domains (six), a Bonferroni-adjusted alpha level of 0.0083 per test was applied to account for multiple comparisons and reduce Type I error risk. Effect sizes (Cohen’s *d*) were calculated for each paired comparison to assess the magnitude of intervention effects (with *d* ≈ 0.2 considered small, ∼0.5 medium, ∼0.8 or above large). It was hypothesized that all domains would show significant gains, reflecting broad improvements in foundational social communication skills.

In addition, the maintenance of treatment effects was examined. We compared the Post-Intervention and Maintenance follow-up scores using paired *t*-tests to see if there was any significant decline or further improvement after the 2-month no-treatment period. A non-significant difference would suggest that gains were maintained. We also compared Maintenance scores to Baseline for completeness, though by design we expected those differences to be similar to the post vs. baseline results. Where relevant, a repeated-measures ANOVA was performed for each skill domain across the three time points (Pre, Post, Follow-up) to provide an omnibus test of change over time; when Mauchly’s test indicated sphericity could not be assumed, Greenhouse-Geisser corrections were applied to the degrees of freedom. These analyses provided a comprehensive view of skill trajectories, confirming whether initial improvements persisted.

Reliability analyses of the quantitative measures were also carried out. Inter-rater reliability was quantified by percent agreement and Cohen’s kappa for each domain (as described in the Materials section), and these values were verified to meet acceptable standards (>0.80) before finalizing the dataset. The high inter-rater consistency indicated that the scoring was dependable. We also examined the internal consistency of the composite skill score at baseline and post-test (Cronbach’s alpha), which remained strong (in the high 0.80 s), suggesting the six domains indeed reflected an underlying construct of “social communication proficiency.”

For the qualitative caregiver feedback, an inductive content analysis approach was used. The post-intervention interviews were transcribed verbatim. Two researchers independently read all transcripts and coded segments of text that reflected common themes or sentiments. The qualitative feedback was ultimately summarized in the results to complement the quantitative findings, providing context and depth. For example, many parents reported increased social engagement from their child (“He’s looking at us and laughing with us so much more now”), improved parent–child interaction quality (“Meal times are more fun because she tries to communicate instead of fussing”), and some challenges (“Sometimes it was hard to fit the sessions into our busy schedule, but it was worth it”). The satisfaction questionnaire data were tabulated and showed high ratings (average >4 out of 5 on most items), which aligned with the generally positive tone of the interviews.

### Method validation

To substantiate the efficacy and reliability of the INSPIRE-Core caregiver-led intervention, a comprehensive validation framework was employed. This included psychometric evaluation of the observational tool, inter-rater agreement analysis, and statistical comparisons of children's pragmatic communication abilities before and after the intervention. A total of 50 toddlers, aged 12 to 36 months, identified with early signs of autism spectrum disorder, participated in the full intervention and assessment protocol.

The findings from the study provided compelling evidence in support of the INSPIRE-Core method as a reliable and effective caregiver-mediated intervention for enhancing social skills early pragmatic communication. The observational checklist developed for assessing progress showed strong psychometric reliability, both in terms of internal consistency and inter-rater agreement, reinforcing its utility as a robust measurement tool. Following the intervention, children demonstrated marked improvements across all six targeted pragmatic domains, including eye contact, social smiling, body posture, joint attention, communication intent, and pointing. These gains were evident not only during structured activities but also within daily home routines, suggesting a meaningful generalization of skills. Notably, children who initially exhibited minimal engagement or limited expressive behaviors began to show clearer social responses, such as initiating interactions, responding to gestures, and sharing attention with caregivers. Caregivers consistently reported increased communication attempts, improved responsiveness, and more enjoyable, interactive routines with their children. Moreover, they rated the program highly in terms of clarity, ease of implementation, and perceived benefit, and expressed confidence in continuing to use the techniques independently beyond the study period. Taken together, these outcomes validate the INSPIRE-Core intervention as both effective and feasible, supporting its broader application in early developmental settings.

### Internal consistency

To evaluate the reliability of the primary outcome measure, internal consistency analysis was conducted on the INSPIRE-Core Pragmatic Skills Checklist, which comprises 12 items—two for each of the six core pragmatic domains. This analysis aimed to assess how well the items functioned together to capture a unified construct of early social-communication competence. Internal consistency analysis was conducted using Cronbach’s alpha, which showed a strong degree of reliability, as evident in Table 4.

**Table 4:** Internal consistency of the INSPIRE-core pragmatic skills checklist.

Timepoint	Cronbach’s Alpha
Pre-Intervention	0.88
Post-Intervention	0.91

The INSPIRE-Core Pragmatic Skills Checklist demonstrated a consistently high level of internal reliability across both points of administration. At the start of the intervention, the Cronbach’s alpha value was 0.88, indicating that the items were already functioning well together in capturing early pragmatic skills. Following the intervention, internal consistency remained strong, with an alpha of 0.91, further confirming the stability and coherence of the tool even after skill changes had occurred.

This pattern of results supports the checklist’s reliability in capturing meaningful variation in social-communication behaviors. The high internal consistency observed at both pre- and post-intervention stages also indicates that the gains made across different domains were not isolated improvements but reflected broader and cohesive progress in children’s pragmatic functioning. The strength and consistency of these scores across time validate the checklist as a dependable tool for evaluating change and progress during early developmental interventions.

### Inter-rater reliability

To ensure objectivity and reproducibility of the assessment process, inter-rater reliability was examined for a randomly selected 20 % of all pre- and post-intervention evaluations. These evaluations were independently scored by two trained raters with expertise in early childhood communication. Both raters underwent a calibration process prior to data collection and were required to reach agreement thresholds on mock scoring tasks before proceeding with study ratings.

The results demonstrated a high level of agreement between raters. Overall percent agreement across the 12 checklist items was 92 %, and the average Cohen’s kappa coefficient was 0.87. As indicated in Table 5, these values demonstrate excellent consistency between raters in their evaluations of children’s pragmatic behaviors during structured and naturalistic interactions.

**Table 5:** Inter-rater reliability of pragmatic skill checklist scoring.

Metric	Value
Percent Agreement	92 %
Cohen’s κ	0.87

The strength of inter-rater reliability across all domains reinforces the robustness of the scoring procedure used in the INSPIRE-Core program. It also confirms that the observed gains were not dependent on subjective interpretation, but rather reflected reliably observable changes in the children’s communication behaviors. These findings further support the methodological soundness of the study and add to the evidence base validating the INSPIRE-Core checklist as a tool for use in both clinical and caregiver-mediated contexts.

### Intervention efficacy

#### Pre–Post comparison

Following the completion of the INSPIRE-Core intervention, notable improvements were observed in children’s pragmatic skills across all six domains assessed. A comparison of pre- and post-intervention checklist scores revealed a substantial overall increase in the average performance level. These gains were evident not only at the group level but also consistent across individual items, suggesting that the improvements were broad-based rather than domain-specific.

Children who initially demonstrated minimal or inconsistent use of early social-communication behaviors—such as limited eye contact, infrequent use of gestures, or poor response to joint attention—began to exhibit these skills more spontaneously and consistently by the end of the program. The largest gains were observed in skills related to intentional communication and joint attention, which are often considered early indicators of social reciprocity. In addition, improvements in more subtle behaviors, such as smiling appropriately and adjusting body posture during interaction, were also documented.

The pre- to post-intervention comparison yielded a highly significant difference in overall checklist scores, indicating that the change observed was unlikely to be due to chance or natural developmental variation alone. The magnitude of the change, as reflected in the effect size, was substantial, supporting the clinical relevance of the INSPIRE-Core approach. Paired sample *t*-tests were used to compare mean scores across the 12 items before and after intervention. The average scores across all items increased significantly, as shown in Table 6.

**Table 6:** Summary of composite pragmatic skill scores at pre- and post-intervention.

Measurement Point	Mean Score	Standard Deviation
Pre-Intervention	1.38	0.13
Post-Intervention	2.76	0.16

These outcomes affirm the effectiveness of the intervention in facilitating real, observable gains in foundational pragmatic abilities over a relatively short period. They also suggest that structured caregiver-mediated support, when delivered consistently and supported with well-designed materials, can produce significant developmental impact even in very young children showing early signs of social-communication difficulties.

The statistical analysis revealed a highly significant difference in children's pragmatic skills before and after the INSPIRE-Core intervention, with a t-value of −75.10 and a p-value <0.001, confirming the reliability of observed changes. The effect size, measured by Cohen’s *d* = 25.21, indicates a very large and meaningful impact, far exceeding conventional thresholds for clinical relevance. These findings underscore not only the statistical strength of the intervention but also its practical efficacy in enhancing early social-communication behaviors.

**Fig. 3:**
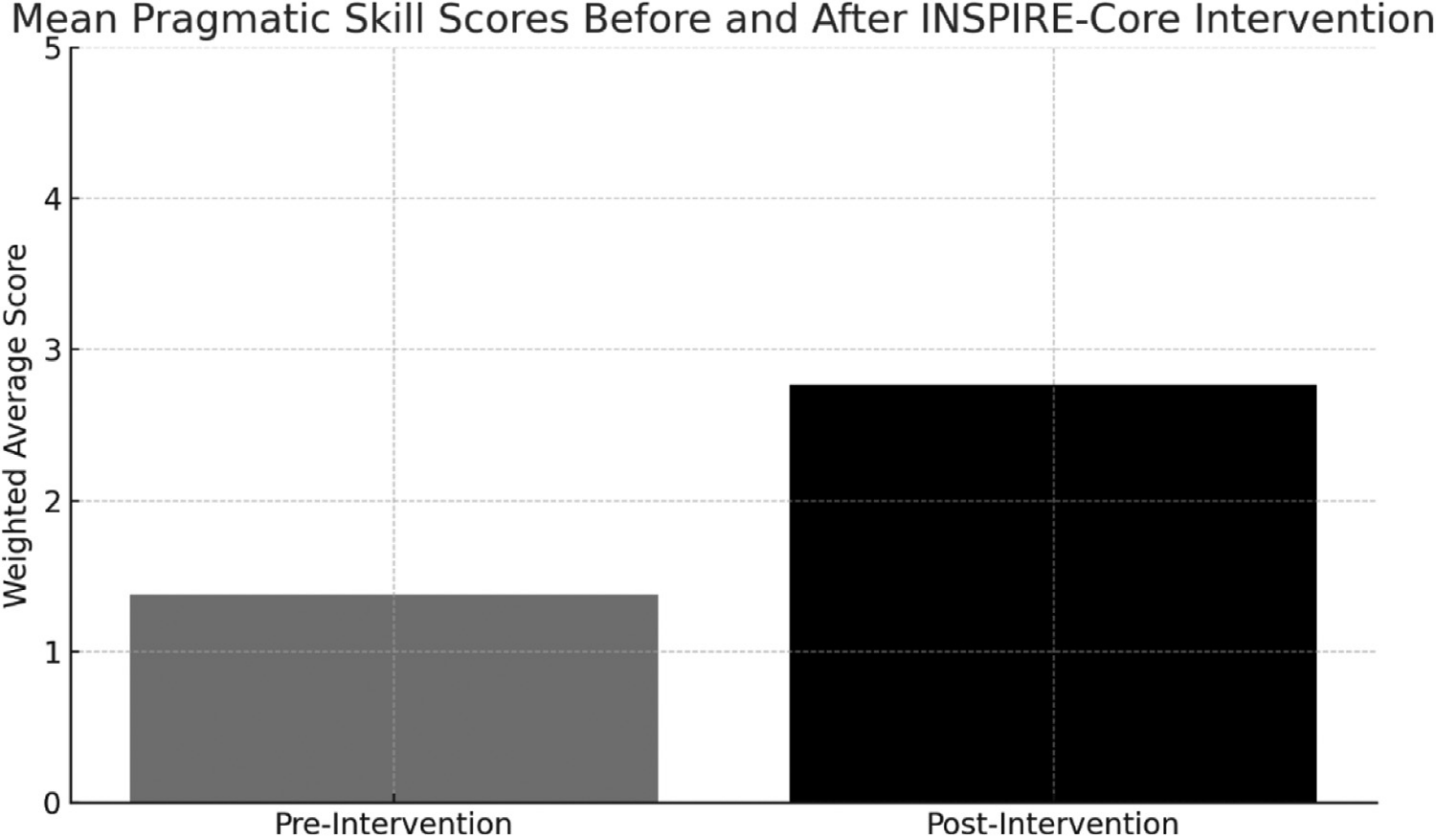
Mean pragmatic skill scores before and after participation in the INSPIRE-Core intervention program.

This change is visually represented in Fig. 3, which illustrates the shift in mean pragmatic skill scores between the pre- and post-intervention phases. As shown, the group’s average score nearly doubled over the course of the intervention, reflecting widespread and consistent developmental gains across the sample.

### Domain-wise item analysis

The INSPIRE-Core intervention resulted in robust improvements across all twelve individual items of the pragmatic skills checklist, representing six foundational domains. Pre- and post-intervention comparisons revealed not only statistically significant changes but also clinically meaningful developmental gains. The increase in scores reflected a clear shift from emerging or prompted behaviors to more frequent, spontaneous, and independent use of early social-communication skills.

In the domain of Eye Contact, both items demonstrated notable improvement. Children progressed from minimal or inconsistent gaze behavior (Pre scores: 1.38 and 1.36) to engaging in eye contact with greater frequency and independence (Post scores: 2.70 and 2.76, respectively). Caregivers noted that children were more likely to seek visual connection during play and social routines, often maintaining gaze without needing explicit prompts. This improvement in mutual gaze laid a strong foundation for later social referencing and interactional awareness.

Social Smiling also showed meaningful gains. The ability to smile in response to social stimuli and as a form of greeting increased from pre-intervention scores of 1.40 and 1.42 to post-intervention scores of 2.78 and 2.80, respectively. These behaviors were often described by caregivers as ``spontaneous'' and “emotionally connected,” representing a shift from passive observation to active participation in social exchanges.

In the domain of Body Posture, both items showed the most marked improvement among the twelve. Scores rose from 1.34 to 1.38 to 2.84 and 2.68, respectively. Children were more frequently observed orienting their bodies toward the speaker, facing their caregiver with openness and readiness to interact. This shift in non-verbal alignment made children’s communicative attempts more visible and effective, especially in shared routines such as storytelling and structured games.

Joint Attention gains were similarly prominent. The ability to show objects to others (Pre: 1.44; Post: 2.74) and maintain shared attention during play (Pre: 1.32; Post: 2.80) improved significantly. These skills are central to the development of shared intentionality and social learning, and their emergence during the program suggests deep engagement with the targeted activities. Activities involving books, bubbles, and peek-a-boo games were particularly effective in fostering these skills.

Within Communication Intent, children displayed increased clarity in initiating interactions and adjusting their messages in response to listener feedback. The ability to request or inform improved from 1.36 to 2.78, while adapting communication strategies based on adult reactions rose from 1.34 to 2.76. These gains were interpreted by caregivers as “more purposeful attempts” to express needs and “more back-and-forth” in daily communication, marking a shift from reactive to interactive behavior.

Pointing—a hallmark of early intentional communication—also improved consistently. The use of pointing to indicate objects or locations increased from 1.42 to 2.78, while the understanding and response to others’ pointing rose from 1.38 to 2.74. These behaviors were frequently observed during daily routines like requesting food items, playing with toys, and identifying pictures in books, and they indicated the children’s growing ability to direct attention and interpret social cues.

As seen in Table 7, across all twelve items, post-intervention scores approached or exceeded the midpoint of the scale, signifying a developmental transition from ``emerging with support'' to ``mostly or fully independent'' in core pragmatic domains. The improvement pattern was consistent across children and domains, demonstrating that the intervention was not only effective in isolated skills but facilitated cohesive, developmental progression in early communication competence.

**Table 7:** Domain-wise comparison of pre- and post-intervention pragmatic skill scores.

Domain	Pre Score	Post Score
Initiates and maintains eye contact	1.38	2.7
Maintains eye contact during conversation	1.36	2.76
Smiles in greeting or response	1.4	2.78
Smiles appropriately to express happiness or agreement	1.42	2.8
Faces speaker with open and attentive posture	1.34	2.84
Maintains physical posturing in conversation	1.38	2.68
Shows objects to share attention	1.44	2.74
Maintains joint attention during play	1.32	2.8
Requests or informs clearly	1.36	2.78
Adjusts communication based on listener feedback	1.34	2.76
Points to indicate object/location	1.42	2.78
Understands/responds to pointing gestures by others	1.38	2.74

### Caregiver feedback and qualitative validation

In addition to quantitative gains, caregiver feedback provided rich insights into the real-world impact, feasibility, and acceptability of the INSPIRE-Core intervention. Following the completion of the program, all participating caregivers completed a structured satisfaction questionnaire and took part in a brief interview to share their experiences. The feedback served not only as a complementary validation source but also as an essential perspective on the program's integration into everyday family life.

Quantitative satisfaction data reflected a high level of approval across all measured dimensions. Caregivers rated the clarity of instructions, child engagement with the materials, ease of home implementation, and overall perceived benefit of the program consistently above 4.6 out of 5. These scores suggest that the toolkit was well-received and manageable for parents with diverse educational and occupational backgrounds.

Thematic analysis of the interview responses revealed three central themes:

**1. Increased Child Engagement and Responsiveness:** Many caregivers described a noticeable shift in their child’s social orientation and willingness to interact. Several reported that their child had become more expressive, engaged more frequently in mutual gaze or turn-taking, and responded more consistently to social cues. Parents noted that simple daily routines such as mealtimes, dressing, and play became more interactive and enjoyable.

“He looks at us now when we call his name, and he even smiles or points to what he wants. Before, it was like we had to guess what he needed.” — Mother of a 2.5-year-old participant

**2. Improvement in Communication Quality**: Caregivers often reported that their child had developed more purposeful communication attempts—using gestures, eye contact, and pointing to express needs. There was a general sense that the child was “trying to connect” more frequently and in more meaningful ways.

“Now she comes to me and shows me things. Earlier, she would just play by herself, but now she tries to include us in what she’s doing.” — Father of a 3-year-old participant

**3. Feasibility and Empowerment in Implementation:** Parents emphasized how the structured yet flexible design of the program allowed them to incorporate activities into their routine without adding significant burden. Several caregivers expressed a sense of confidence in using the strategies independently after the program ended and appreciated that the activities did not require specialized materials or clinical environments.

“The best part was that we could do this at home. The storybooks made it fun, and I didn’t feel lost. I knew exactly what to do, and it didn’t feel like therapy—it felt like play.” — Caregiver of a 2-year-old participant

While a few caregivers mentioned occasional difficulties in maintaining session regularity due to competing demands or the child’s mood on certain days, these were typically short-lived and were effectively addressed during bi-weekly coaching calls or home visits.

Overall, caregiver responses were overwhelmingly positive, which is reflected in the satisfaction ratings summarized in Table 8. The average rating for clarity of instructions was 4.8, while perceived benefit was rated the highest at 4.9, indicating that families found the intervention not only understandable and accessible but also highly valuable. Engagement value for the child [[4], [5], [6]] and ease of implementation at home [[4], [5], [6], [7]] also received strong endorsements, further affirming the program’s practical applicability in naturalistic caregiving settings.

**Table 8:** Caregiver satisfaction ratings following INSPIRE-core intervention.

Feedback Dimension	Average Rating (out of 5)
Clarity of Instructions	4.8
Engagement Value for Child	4.6
Ease of Use at Home	4.7
Perceived Benefit	4.9

The INSPIRE-Core intervention demonstrated strong evidence of effectiveness and reliability through both quantitative outcomes and caregiver-reported improvements. The consistent domain-wise gains, high satisfaction, and practical feasibility position INSPIRE-Core as a validated method for enhancing early pragmatic communication in toddlers at risk for ASD.

### Maintenance and follow up

At the conclusion of the two-month maintenance period, a follow-up evaluation was conducted to assess whether the gains observed at post-intervention had been retained. The scoring procedures mirrored those used at baseline and post-intervention, allowing for direct comparisons across all three time points. Caregivers also reported on the continued presence of pragmatic skills in everyday contexts and any new environmental influences (e.g., daycare entry, speech therapy enrollment).

**Fig. 4:**
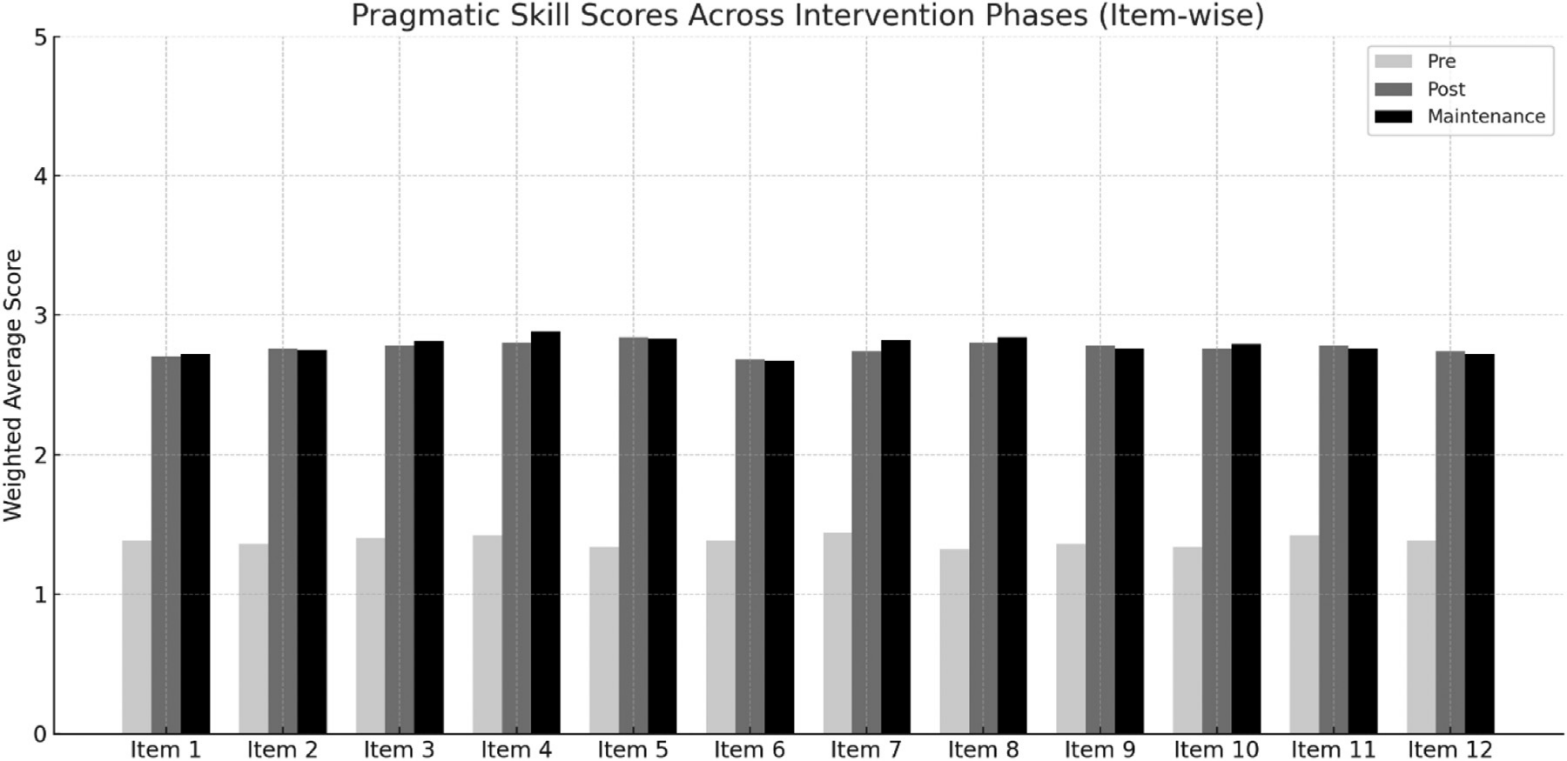
Item-wise comparison of pragmatic skill scores across intervention and maintenance phases.

As evident from Fig. 4, the statistical analyses revealed no significant decline in performance between the post-intervention and follow-up assessments across any of the six pragmatic domains. Paired *t*-tests confirmed that skill levels remained stable, indicating strong maintenance of intervention effects over time. Comparisons between the baseline and follow-up scores also remained significant, suggesting that the skills acquired during the intervention were not only retained but remained elevated relative to initial functioning.

### Limitations

• While a control group was not included in this phase, the consistent pre–post–maintenance progression supports the potential efficacy of the intervention and lays the foundation for future controlled trials.
• The current sample, although drawn from a specific region, provided valuable insight into caregiver-led implementation in real-world Indian home settings; further studies can extend these findings to more diverse populations.
• The use of checklist-based observations and caregiver input offered practical, context-sensitive evaluation; future work may incorporate additional standardized tools or blinded assessments to complement these rich data sources.
• Cultural and linguistic alignment were prioritized in the toolkit’s development, though broader validation across varied sociocultural backgrounds would strengthen its adaptability.
• A two-month maintenance phase was feasible and sufficient for this early pilot; longer-term tracking is planned to explore sustained impact over time.

## Declaration of interests

The authors declare that they have no known competing financial interests or personal relationships that could have appeared to influence the work reported in this paper.

## Data Availability

The data that has been used is confidential.
